# Genetic affinities of six populations of Manipur using a microsatellite (STR) marker

**DOI:** 10.1186/1755-8166-7-S1-P107

**Published:** 2014-01-21

**Authors:** Ahsana Shah, Ruqaiya Hussain, Mohammad Afzal

**Affiliations:** 1 Aligarh Muslim University, Aligarh, U.P., India

## Background

The development of molecular genetic technology has led to the discovery of large number of polymorphic loci in the human genome. This has renewed the interest in investigating genomic diversity (and affinity) between human populations with much more depth and clarity than that was possible with the erstwhile traditional serological and biochemical genetic markers. DNA markers are presently widely used in gene mapping, forensic studies and information on population structure and population genetics of regional and global populations. The study attempts to understand the genetic structure and affinity among six different populations of Manipur.

## Materials and methods

The diversity of the studied populations was examined by investigating the polymorphism for a STR locus (TPOX). Unrelated individuals belonging to Muslims with different caste viz. Sheikh, Syed, Pathan & Moghul; Hindu (Meitei) and tribal (Naga) were randomly selected. Blood samples were collected after obtaining an informed consent. The DNA was extracted using standard phenol – chloroform extraction method and then amplified with PCR using previously published primers. The amplified PCR products were electrophoreses followed by Silver staining.

## Results

Different population containing allele with repeated number varying from 5 -11 were detected. Discriminating Power Analysis (PD) lie in the range from 0.444 to 0.889 and Matching Probability (MP) was observed to vary from 0.111 to 0.556. Based on genetic distance, dendrogram was constructed to depict the genetic affinities among the six populations. The tree shows that the ethnic background of six populations is different.

